# Cell Cycle-Dependent Induction of Homologous Recombination by a Tightly Regulated I-SceI Fusion Protein

**DOI:** 10.1371/journal.pone.0016501

**Published:** 2011-03-09

**Authors:** Andrea Hartlerode, Shobu Odate, Inbo Shim, Jenifer Brown, Ralph Scully

**Affiliations:** Department of Medicine, Harvard Medical School and Beth Israel Deaconess Medical Center, Boston, Massachusetts, United States of America; University of Pennsylvania, United States of America

## Abstract

Double-strand break repair is executed by two major repair pathways: non-homologous end joining (NHEJ) and homologous recombination (HR). Whereas NHEJ contributes to the repair of ionizing radiation (IR)-induced double strand breaks (DSBs) throughout the cell cycle, HR acts predominantly during the S and G2 phases of the cell cycle. The rare-cutting restriction endonuclease, I-SceI, is in common use to study the repair of site-specific chromosomal DSBs in vertebrate cells. To facilitate analysis of I-SceI-induced DSB repair, we have developed a stably expressed I-SceI fusion protein that enables precise temporal control of I-SceI activation, and correspondingly tight control of the timing of onset of site-specific chromosome breakage. I-SceI-induced HR showed a strong, positive linear correlation with the percentage of cells in S phase, and was negatively correlated with the G1 fraction. Acute depletion of BRCA1, a key regulator of HR, disrupted the relationship between S phase fraction and I-SceI-induced HR, consistent with the hypothesis that BRCA1 regulates HR during S phase.

## Introduction

Repair of mammalian chromosomal double-strand breaks (DSBs) entails use of one of two major repair pathways: non-homologous end-joining (NHEJ) and homologous recombination (HR) (reviewed in [1], [2], [3], [4]). These repair functions take place in relation to other chromosome processes such as transcription and DNA replication, and DNA replication alters the parameters governing DSB repair in several ways. First, the process of DNA replication itself is thought to be a major cause of endogenous DSBs. Second, replication generates a second copy of the chromosome in the form of a sister chromatid, which may favor DSB repair by homologous recombination, (“sister chromatid recombination” – SCR) [5], [6], [7], [8]. Third, cell cycle-dependent variations in the activity of cyclin-dependent kinases affect the likelihood of engaging the DSB processing enzymes required for HR [9]. In budding yeast, where HR is the major DSB repair pathway, repair of ionizing radiation (IR)-induced DSBs engages SCR in preference to interhomolog recombination [7]. Similarly, in mammalian cells, intrachromosomal HR (of which SCR is likely a major component) is more efficient than interchromosomal HR [10]. In contrast to the constraints on HR, NHEJ can, in principle, operate at any stage of the cell cycle.

Studies in which IR was used to induce chromosome breakage have supported the idea that HR and NHEJ contribute differently to DSB repair at different stages of the cell cycle [11], [12], [13]. In the chicken lymphoblastoid cell line, DT40, cells defective for the HR gene *RAD54* reveal increased IR-sensitivity specifically in S phase [14]. In contrast, DT40 cells lacking the NHEJ gene *KU70* are hypersensitive to IR during G1 and early S phase, consistent with a major role for NHEJ in repair of breaks generated prior to replication. Similar cell cycle dependencies of IR sensitivity and IR-induced HR were observed in mammalian cells, further supporting the notion that IR-induced HR is limited to the S/G2 cell cycle phases [12]. Indeed, in cells lacking the NHEJ factor XRCC4, IR-induced DSBs generated in G1 triggered elevated levels of HR in the subsequent S/G2. This suggests that the mechanism engaged to repair a DSB is a function of the cell cycle stage at which repair is executed, rather than the stage at which the DSB itself is induced [11], [13].

The rare-cutting restriction endonuclease, I-SceI, has become a major tool for analyzing mechanisms of DSB repair [15]. I-SceI-induced breaks are likely limited to the canonical I-SceI target site and the DSB ends generated by I-SceI-mediated breakage are defined by the enzyme's known endonuclease activity. In contrast, IR-induced DSBs are distributed across the genome and the DSB ends may be chemically modified [16]. For these reasons, the “rules” governing I-SceI-induced DSB repair might differ from those governing an IR-induced DSB. The ligand-binding domain (LBD) of the estrogen receptor (ER) has been used to regulate the activity of a variety of nuclear proteins, since fusion proteins containing the ER LBD are retained in the cytoplasm until activated by 17ß-estradiol [17], [18], [19]. Ligand binding facilitates correct folding of the released fusion protein, thereby activating the LBD-fused nuclear protein. The mouse ER LBD mutant G525R exhibits a ∼1000-fold reduction in 17ß-estradiol binding affinity compared to the wildtype protein, but retains normal affinity for the synthetic ER ligands and functional estrogen antagonists, tamoxifen (TAM) and 4-hydroxy-tamoxifen (4OHT) [20]. The corresponding human ER LBD mutant (G521R, termed “ER^T^”) has similar properties [17]. The 4OHT affinity of the human ER LBD mutant ER^T2^ (G400V/M543A/L544A) is approximately 4-fold higher than that of ER^T^ [21]. Ectopic DNA enzymes such as Cre, Flp and I-SceI have successfully been controlled by fusion with ER^T^ [21], [22], [23], or with other nuclear hormone receptors [24]. In work described here, we characterize a set of I-SceI-ER^T^ or ER^T2^ fusion proteins and define optimal configurations for tight control of I-SceI activity, as measured by a sensitive I-SceI-inducible HR reporter. By enriching cells in specific phases of the cell cycle prior to activation of I-SceI, we have used this tool to study the impact of cell cycle stage on I-SceI-induced HR.

## Materials and Methods

### Plasmids and RNAi

Expression plasmids for ER^T^ and ER^T2^ were described previously [21]. New constructs described here were generated by standard cloning procedures. Control RNAi duplex against luciferase (5′-CGUACGCGGAAUACUUCGAdTdT-3′) and RNAi duplex against *BRCA1* (5′-AAUCACAGUGUCCUUUAUGUAdTdT-3′) were purchased from Dharmacon.

### Cell Lines and Cell Culture

U2OS SCR #18 cells were described previously [25]. ER^T^ stable lines were maintained in phenol red-free DMEM supplemented with 10% charcoal-stripped fetal bovine serum (CS-FBS, Atlanta Biologicals), 0.2 ng/mL EGF (Invitrogen) and 100 U penicillin/100 mg streptomycin at 37°C and 6% CO_2_. To generate ER^T^ stable lines, the various ER^T^ expression constructs were transfected into U2OS SCR #18 cells and 0.4 mg/mL G418 (Sigma-Aldrich) was added to the medium 2 days after transfection. After 2 weeks under continuous selection, G418-resistant colonies were isolated and screened by flow cytometry for 4OHT-induced HR.

### Antibodies and Immunoblotting

Cells were lysed in NP-40 lysis buffer (50 mM Tris-HCl [pH 8.0], 1% NP-40, 150 mM NaCl) supplemented with protease inhibitor cocktail (Roche). Cell lysates were resolved by SDS-PAGE on NuPAGE® Novex Bis-Tris Gels (Invitrogen) and analyzed by blotting with mouse monoclonal anti-Myc (9E10) antibody.

### Immunofluorescence Staining

Cells on glass coverslips were treated with 1 µM 4OHT for the desired length of time (0–180 min), fixed in 3% paraformaldehyde/2% sucrose, permeabilized in Triton X-100 solution (0.5% Triton X-100, 20 mM HEPES [pH 7.4], 50 mM NaCl, 3 mM MgCl_2_, 300 mM sucrose), stained with anti-Myc (9E10) followed by goat anti-mouse IgG rhodamine-conjugated secondary antibody and imaged on a Zeiss microscope.

### Recombination Assays

Cells were plated at a density of 0.4×10^6^ cells/well on 6-well plates overnight prior to assay. Cells were treated with 1 µM 4OHT (Sigma-Aldrich) and GFP^+^ frequencies were measured 24–72 hr post-treatment by flow cytometry using an FC500 (Beckman Coulter).

### Cell Cycle Arrest

In order to arrest/synchronize cells at different stages of the cell cycle, several drug treatments were used. For G2/M synchronization, cells were treated for 16 hrs in 0.4 ng/mL nocodazole (Sigma-Aldrich), harvested by mitotic shake-off, and plated at a concentration of 0.4×10^6^ cells/well on 6-well plates with or without 1 µM 4OHT for 48 hrs. For G1 arrest by double drug treatment, cells were treated for 16 hrs in 0.4 ng/mL nocodazole (Sigma-Aldrich), harvested by mitotic shake-off, and plated at a concentration of 0.4×10^6^ cells/well on 6-well plates in 40 mM lovastatin (Axxora) for 48 hrs. 4OHT was added at a final concentration of 1 µM to induce I-SceI at the same time as cells were plated into lovastatin. For G1 arrest by single drug treatment, 40 mM lovastatin was added to cells with or without 1 µM 4OHT for 48 hrs.

### Cell Cycle Profiling

Cells were pulsed with 10 µM bromodeoxyuridine (BrdU, Sigma-Aldrich) for 20 min, collected, and fixed with ice cold 70% ethanol. For staining cells were treated with 2 M HCl/0.5% Triton X-100, neutralized with 0.1 M Na_2_B_4_O_7_ (pH 8.5), stained with mouse anti-BrdU primary antibody (Boehringer-Mannheim) and then goat anti-mouse IgG FITC-conjugated antibody (Jackson ImmunoResearch). Finally, cells were resuspended in 38 mM sodium citrate/69 µM propidium iodide/100 µg/mL RNaseA and propidium iodide/FITC staining levels were measured by flow cytometry using an LSR II (BD Biosciences).

### Southern Blotting

Genomic DNA was extracted from 5–10×10^6^ cells using the ArchivePure Cell/Tissue Kit (5 PRIME). Southern blotting for wt*GFP* was performed as described previously [25].

### Transfection

0.5×10^6^ U2OS SCR NEIE were plated overnight then transfected with 120 pmol siRNA using Lipofectamine™ 2000 (Invitrogen). GFP^+^ frequencies were measured 72 hr post-transfection by flow cytometry using an LSR II (Beckman Coulter).

## Results

**Figure 1:**
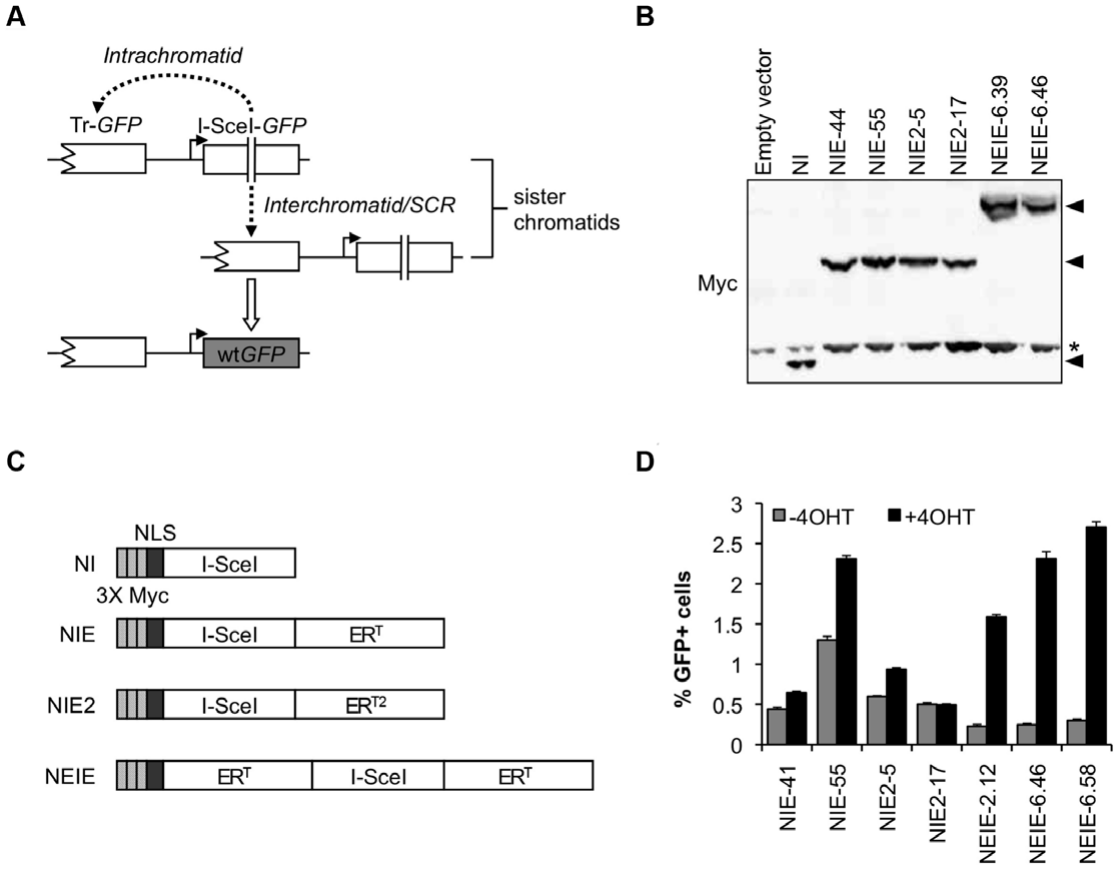
Optimization of I-SceI-ER^T^ fusion proteins for controlled induction of chromosome breakage. A) Repair of an I-SceI-induced DSB by intra- or interchromatid gene conversion. Double vertical lines: restriction site cut by I-SceI; Solid arrow: promoter sequence. B) I-SceI expression constructs used in this study. Each construct has an N-terminal triple-Myc tag and nuclear localization signal. ER^T^ represents the G521R mutant and ER^T2^ represents the mutant containing the G400V/M543A/L544A triple mutation of the human estrogen receptor ligand-binding domain. NI – NLS-I-SceI; NIE – NLS-I-SceI-ER^T^; NIE2 – NLS-I-SceI-ER^T2^; NEIE – NLS-ER^T^-I-SceI-ER^T^. C) Stable expression of I-SceI fusion proteins. Asterisk indicates endogenous Myc protein and can be used as a loading control. Arrowheads indicate predicted sizes of I-SceI fusion proteins. D) 4OHT-induced HR in NIE, NIE2, and NEIE cell lines. Error bars represent the standard error of the mean (S.E.M.) for triplicate samples.

We used a previously described HR reporter containing two tandem mutant copies of the gene encoding the enhanced green fluorescent protein (*EGFP*, here termed *GFP*) (**Figure 1A**) [25], [26]. The first *GFP* copy (Tr-*GFP*) is truncated at the 5′ end and the second *GFP* copy (I-SceI-*GFP*) is full length, but is interrupted by the 18 base pair (bp) recognition site for the rare-cutting endonuclease, I-SceI, rendering this copy nonfunctional. Expression of I-SceI induces a site-specific DSB within the reporter and stimulates HR. Recombination between the two *GFP* copies by either intra- or inter-chromatid HR (**Figure 1A**), but not single strand annealing, can generate wild type *GFP* (wt*GFP*), which is readily quantified by flow cytometry.

We fused cDNA sequences encoding ER mutants, ER^T^ or ER^T2^, in frame with the 3′ end of I-SceI in the I-SceI expression construct pcDNA3ß-myc-NLS-I-SceI, to generate “NIE” and “NIE2” constructs respectively (**Figure 1B**). We generated U2OS HR reporter cell lines (U2OS #18 [25]) stably transfected with either of these constructs and screened individual clones for the induction of HR upon addition of 4OHT (1 µM) to the medium. Steady state levels of the fusion proteins are shown in **Figure 1C**. 4OHT-responsive clones were identified for each construct; however, I-SceI-ER^T^ generated a higher proportion of responsive clones than I-SceI-ER^T2^. In clones that were found to be functionally responsive, as measured by induction of GFP^+^ events by 4OHT, the addition of 4OHT also produced the expected nuclear accumulation of myc-tagged I-SceI-ER^T^ or -ER^T2^ fusion protein. However, despite cultivation of the cells in phenol red-free medium and charcoal-stripped serum, every responsive clone revealed variable, weakly positive nuclear immunostaining for myc during passage in the absence of 4OHT. Further, all NIE or NIE^2^ 4OHT-responsive clones revealed an accumulation of GFP^+^ events in the absence of 4OHT at rates well above that observed in the parental clones lacking I-SceI-ER^T^ or -ER^T2^ expression (**Figure 1D** and data not shown). This indicates that the NIE and NIE^2^ fusion proteins were not perfectly controlled and that I-SceI-mediated breakage occurred even in the absence of the inducing 4OHT ligand.

It was reported previously that the addition of more than one ER^T^ domain can improve the background of Cre fusion proteins [18]. We therefore generated an I-SceI-ER^T^ fusion protein in which a second ER^T^ domain was fused, in frame, between the NLS and I-SceI sequences of NIE, to generate a construct encoding a myc-NLS-ER^T^-I-SceI-ER^T^ fusion protein (here termed “NEIE” – **Figure 1B**). Following stable expression of NEIE in U2OS HR reporter cell line #18 (**Figure 1C**), we identified 4OHT-responsive clones and observed that, when cultivated in phenol red-free medium and charcoal-stripped serum, 4OHT-responsive NEIE clones appeared to exhibit improved stability in the absence of 4OHT than either NIE or NIE2 clones, while retaining equivalent responsiveness in the presence of the ligand (**Figure 1D**). The majority of 4OHT-responsive NEIE clones revealed a persistently low background frequency of GFP^+^ events, while none of the 4OHT-responsive NIE or NIE2 clones showed this pattern. Representative clones of each type are shown in **Figure 1**. While this work was in progress, Bennardo *et al.* reported similar observations using an independently generated system [23].

**Figure 2:**
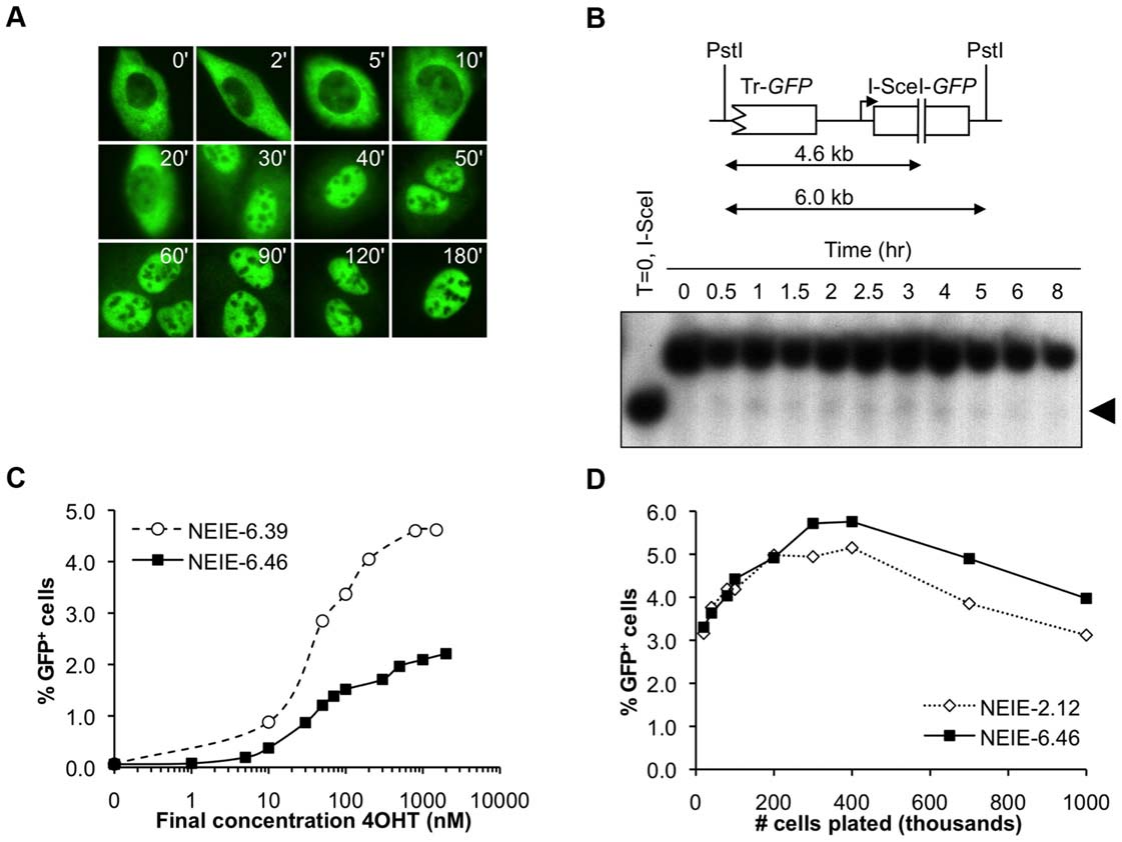
Time course and dose dependence of HR induced by 4-hydroxytamoxifen in NEIE cells. A) Immunofluorescence detection of 4OHT-induced NEIE nuclear accumulation over time. NEIE-6.46 cells were treated with 4OHT for the indicated times (in minutes) before fixation and staining. B) Southern blot detection of a 4OHT-induced site-specific DSB within the HR reporter. Arrowhead indicates the migration of PstI-restricted gDNA that has been cut by I-SceI. The uncut band is not shown in this figure. C) The effect of 4OHT concentration on NEIE-induced HR. The average raw percentage of GFP^+^ cells within the culture is shown. D) The effect of cell density on NEIE-induced HR. Shown is the average percentage of GFP^+^ cells, corrected for the background level of GFP^+^ cells.

To further characterize the performance of the NEIE fusion protein, we used immunostaining for myc followed by immunofluorescence microscopy to study the subcellular distribution of the NEIE fusion protein either in the absence of 4OHT or at defined times following addition of 4OHT to the medium (**Figure 2A**). Notably, in the absence of 4OHT, the NEIE fusion protein was observed in the cytoplasm and was fully excluded from the nucleus. Within 10 to 20 minutes of 4OHT addition, the NEIE protein was found to accumulate in the nucleus, and by 1 hour nearly every cell contained fully nuclear-localized NEIE protein.

To test whether the freshly nuclear relocalized NEIE protein was active, we sought evidence of chromosome breakage within the HR reporter during the 4OHT response. We harvested genomic DNA (gDNA) from clone NEIE-6.46 either before the addition of 4OHT, or at defined time points following 4OHT addition, and analyzed the structure of the HR reporter by Southern blotting, as described in Materials and Methods. Briefly, PstI-digested gDNA fragments that are probed with *GFP* reveal a single fragment of ∼5.9 kb if the reporter locus is intact. If the I-SceI site has been cut, a lower molecular weight species of ∼4.6 kb is observed (**Figure 2B**). Only a very small fraction (<1%) of cells at any given time point following 4OHT addition revealed the I-SceI-cut ∼4.6 kb band (**Figure 2B**). The I-SceI-cut fragment was noted within 30 minutes following 4OHT addition and its intensity was approximately constant over time (**Figure 2B**). This suggests that the NEIE fusion protein is functionally active at the time of nuclear entry. Further, the low but consistent level of I-SceI-induced breakage observed at all time points following 4OHT addition suggests that there is a dynamic equilibrium between I-SceI-mediated chromosome breakage and DSB repair, with the balance tipped heavily in favor of repair in the cells examined here. This might reflect efficient religation of the I-SceI-induced DSB by precise NHEJ, which accurately reconstitutes the I-SceI site, provided canonical NHEJ is intact [27], [28]. However, in other experiments in which we examined breakage at the I-SceI site using Southern blotting in *Ku70* ^−/−^ or *XRCC4* ^−/−^ mouse embryonic stem cells, we failed to observe dramatic increases in steady state levels of I-SceI breakage, despite the loss of the canonical NHEJ pathway (data not shown). This might reflect cell type-specific effects or the engagement of alternative error-free DSB repair pathways. Further, the activity of the I-SceI endonuclease might be reduced in the context of the fusion protein.

Importantly, removal of 4OHT from the culture medium of induced NEIE cells did not disrupt the nuclear distribution of the activated NEIE protein, and the strong nuclear myc signal persisted for up to 12 hours (data not shown). Therefore, although 4OHT can be used to activate I-SceI with precise timing, withdrawal of 4OHT appears not to immediately inactivate the enzyme.

The above experiments were performed with 1 µM 4OHT calculated to saturate LBD receptor occupancy. To confirm that this dose was appropriate, we generated a dose-response curve for two of the NEIE stable cell lines, NEIE-6.39 and NEIE-6.46 (**Figure 2C**). I-SceI-induced HR in each test sample was assayed as the percentage of 4OHT-induced GFP^+^ cells. The resultant dose-response curves revealed median effective doses (ED_50_) of approximately 40 nM for each cell line - values similar to that reported previously for a Cre-ER^T^ fusion protein [21]. 4OHT-induced HR in each cell line showed a modest dependence on the plating density of cells (**Figure 2D**).

**Figure 3:**
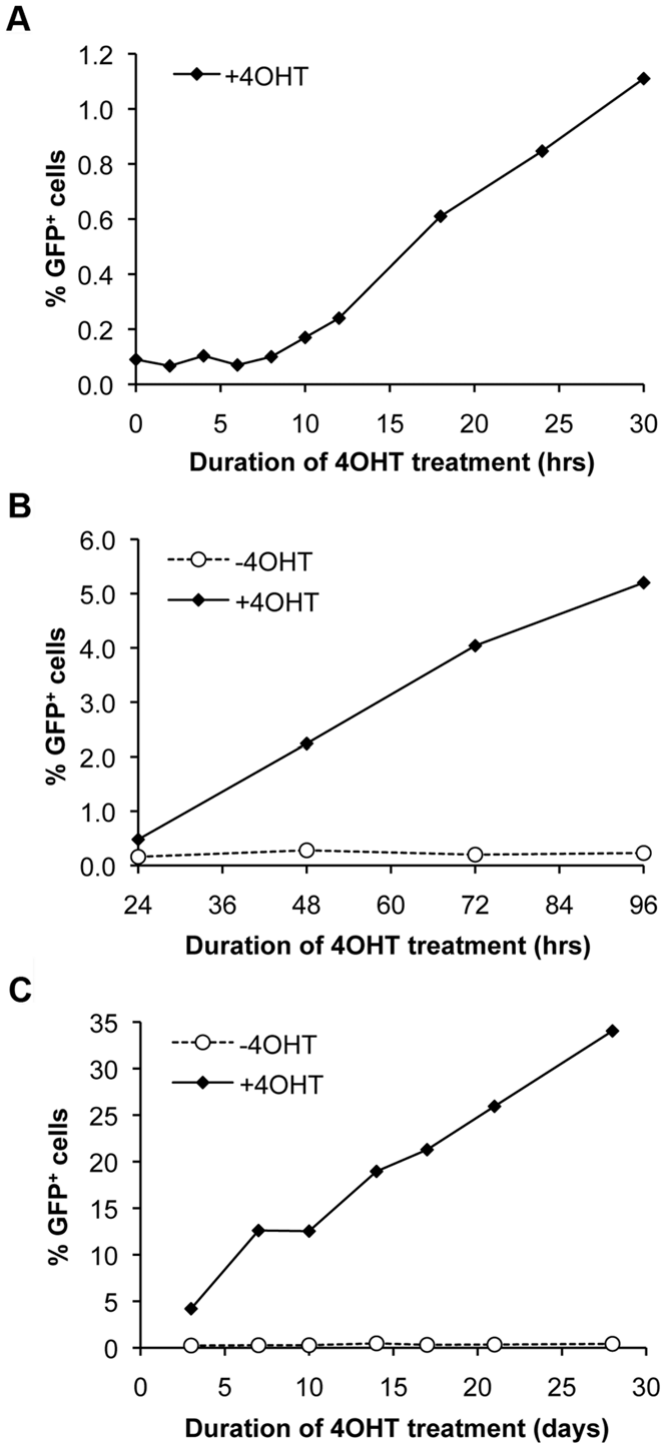
I-SceI activation induces a fixed rate of HR and GFP^+^ cell accumulation. A) Initial emergence of GFP^+^ cells in NEIE-6.46 cells occurs after 10 hrs in 4OHT. B) The frequency of GFP^+^ cells increases in a linear fashion over the normal time course of experiments in this study (72 hrs). C) Linear increase in the frequency of GFP^+^ cells continues in cells exposed to 4OHT for up to 28 days. Open circle: no 4OHT. Black triangles: with addition of 4OHT.

To determine the temporal relationship between DSB induction and HR, we examined the emergence of GFP^+^ cells as a function of duration of treatment of cell line NEIE-6.46 in 4OHT (1 µM). GFP^+^ cells first became detectable by flow cytometry within 10 to 12 hours of 4OHT addition (**Figure 3A**). Thereafter, GFP^+^ cells accumulated in 4OHT-treated populations at a fixed rate (**Figure 3B**). This rate of increase of the GFP^+^ fraction was remarkably constant at ∼0.05% per hour (∼1.2% per day), even when cells were passaged in 4OHT for a period of up to one month (**Figure 3C**). In contrast, cells passaged in the absence of 4OHT showed no increase in the GFP^+^ fraction over this one-month period, indicating that the NEIE cell line is tightly regulated when cultivated in medium lacking estrogenic compounds.

**Figure 4:**
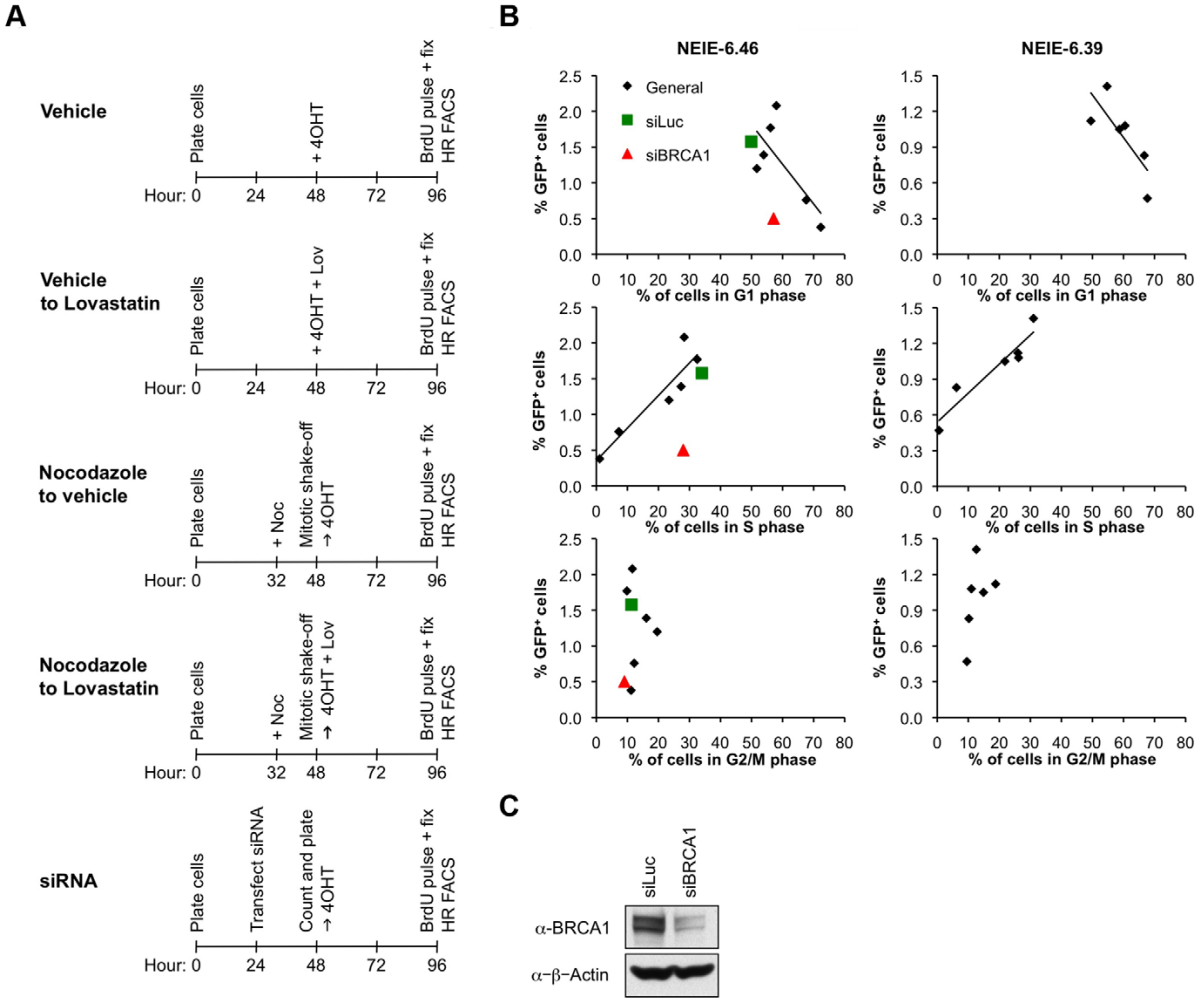
BRCA1 depletion disrupts HR in S phase. A) Cell synchronization methods. B) U2OS HR NEIE-6.39 and 6.46 cell lines were used to determine the cell cycle profile and frequency of HR after 48 hrs of 4OHT exposure in cultures synchronized at different stages of the cell cycle. Black circles represent data from several experiments involving different drug treatment protocols, as described in Materials and Methods. These treatments include vehicle only, Lovastatin (Lov) alone, and cells treated with Nocodazole (Noc) and released into either vehicle or Lovastatin. A strong positive correlation is noted between the percentage of cells in S phase and the efficiency of HR, as indicated by the line of best fit (NEIE-6.39 *y* = 0.0244*x*+0.5395, R^2^ = 0.89503; NEIE-6.46 *y* = 0.0456*x*+0.3533, R^2^ = 0.85193). A negative correlation between the percentage of cells in G1 phase and the efficiency of HR is also noted (NEIE-6.39 *y* = −0.0357*x*+3.1208, R^2^ = 0.61909; NEIE-6.46 *y* = −0.0555*x*+4.588, R^2^ = 0.52032). The U2OS HR NEIE-6.46 cell line was transfected with control siRNA (siLuc – siLuciferase) or BRCA1 siRNA (siBRCA1). 24 hrs post-transfection cells were replated in either 1 µM 4OHT or vehicle control, for a further 48 hours. Green squares indicate siLuc data points and red triangles indicate BRCA1 siRNA data points. C) Immunoblotting for BRCA1 in different siRNA treated groups.

We asked whether I-SceI-induced HR is influenced by the cell cycle stage during which I-SceI is activated. To study this, we used combinations of lovastatin and nocodazole to synchronize cells at various points in the cell cycle. Lovastatin inhibits HMG-CoA reductase, causing cells to arrest in G1 [29], while nocodazole is an antimitotic agent that disrupts microtubules and arrests the cell cycle at the G2/M phase [30]. Importantly, these drugs are not predicted to have a direct impact on DSB repair. Cell synchronization methods are depicted **Figure 4A**. In all treatment groups, cells were plated at the start of the experiment, were exposed to 4OHT 48 hours later, and were harvested for analysis of HR (measured by induction of GFP^+^ cells) and cell cycle distribution at the 96 hour time-point (i.e., 48 hours after initial exposure to 4OHT). Note that cells were exposed to 4OHT continuously throughout the second 48 hour period. Upon this framework we imposed one or more of the following two treatments: first, Nocodazole treatment for 16 hours, followed by release from nocodazole by mitotic shake-off, washing and replating, with the replating timed to coincide with the addition of 4OHT (the 48 hour time point). Second, lovastatin treatment, added to the medium at the same time as 4OHT (i.e., the 48 hour time point) and left in the medium until the time of harvesting (96 hour time point). Four treatment groups were analyzed: one received control diluents (“vehicle”) but no cell cycle synchronizing drug; a second received nocodazole synchronization only; a third received lovastatin only; and a fourth received nocodazole+lovastatin (**Figure 4A**). As expected, lovastatin or nocozazole alone each diminished the percentage of cells in S phase at the end point of the experiment, and this effect was enhanced if the two drug treatments were combined (**Supplemental Table S1**).

To determine whether cell cycle status at the time of harvest (i.e., the 96 hour time point) had any predictive value for the efficiency of HR measured at that same time point, we plotted the percentage of I-SceI-induced GFP^+^ cells against the percentage of cells in G1, S or G2 phases of the cell cycle, combining data from the four different treatment groups into one graph (**Figure 4B**). We generated regression lines for each of two independent clones (NEIE-6.39 and NEIE-6.46) and calculated the optimal fit of regression lines for each combination by the least squares method (**Figure 4B**). This analysis revealed a strong positive linear correlation (R^2^ = 0.90 and 0.85 for NEIE-6.39 and NEIE-6.46, respectively) between the percentage of cells in S phase and the level of 4OHT-induced HR (**Figure 4B**). We also observed a weak negative correlation (R^2^ = 0.62 and 0.52) between the percentage of cells in G1 and the efficiency of 4OHT-induced HR. These results show that I-SceI-induced HR in this system is strongly correlated with S phase, and that HR is inefficient in G1. This suggests that I-SceI induces a cell cycle-dependent pattern of HR equivalent to that deduced from previous analysis of IR-induced DSB repair [11], [12], [14]. However, some caution is needed in interpreting our results. First, the nature of the cell cycle synchronization is not absolute; it is to be expected that cell cycle distribution will vary over the 48 hours of 4OHT treatment, as cell cultures encounter the G1 block imposed by lovastatin or traverse G1 and enter S, in the case of the release from nocodazole. Second, we cannot exclude the possibilty that I-SceI-mediated DSB induction is itself cell cycle regulated. With these caveats in mind, the data does appear to support the notion that, operationally, I-SceI-induced HR is strongly correlated with S phase.

BRCA1 is known to interact with Rad51 in discrete nuclear foci during the S and G2 phases of the cell cycle [31] and to relocalize rapidly to sites of replication arrest in S phase cells treated with HU [32]. In addition, *BRCA* mutant embryos contain chromosomes with “chromatid-type” structural aberrations, which are considered to arise from replication across a damaged template and from recombination errors in S phase [33], [34]. These observations led to the proposal that BRCA1 regulates SCR during S phase [35]. To test this hypothesis, we transfected U2OS HR NEIE cells with control siRNA or siRNA directed against human BRCA1, and plated cells directly into 4OHT 24 hours post-transfection, harvesting cells 48 hours later (**Figure 4A and 4C**). Depletion of BRCA1 reduced 4OHT-induced HR in U2OS HR NEIE cells, in comparison to control siRNA, but with minimal disruption of the cell cycle profile (**Figure 4B**). These results suggest that loss of BRCA1 disrupts the relationship between of S phase fraction and HR, and therefore support the long-standing hypothesis that BRCA1 has a major influence on HR during S phase.

## Discussion

The development and characterization of a system to control I-SceI expression in a tight temporal fashion is an important research tool for the study of DSB repair. Its applications may include the study of cell cycle relationships, as discussed above, to potential use in chromatin immunoprecipitation studies [36], real-time imaging, high-throughput screening and tight control of I-SceI activity in animal models. The accurate interpretation of siRNA experiments might also benefit from a tractable inducible DSB system such as that described here, where DSB induction could be initiated when siRNA knockdown is at its peak. The use of a recombination reporter is a stringent test of the activity of the enzyme – probably more so than attempts to directly measure breakage at the locus. Notably, cultivation of cells in the presence of phenol red and endogenous estrogens in serum that was not charcoal stripped, triggered the gradual accumulation of GFP^+^ products of I-SceI-induced HR in the absence of 4OHT (data not shown). This suggests that, if residual estrogens are present in the culture medium, they can produce some basal activation of the fusion protein, even in its optimal “EIE” configuration. This suggests that the application of this technology in animal models may require additional levels of control.

In this report, we have identified an optimal configuration of ER-LBD-I-SceI fusion protein that provides tight control of I-SceI activity in the absence of activating ligand. Our experience matches closely that described for ER LBD control of the Cre recombinase [18] and the results of Bennardo *et al.* in control of I-SceI [23]. In each, the addition of ER LBD domains to both the N- and C-termini of the enzyme provide optimal control with retention of enzymatic activity. This result is also consistent with the crystal structure of I-SceI, in which the active site is predicted to be unaffected by modification of either the N- or C-termini of the polypeptide [37].

We used this construct, stably expressed in U2OS HR reporter cells, to study the relationship between cell cycle stage and HR, by activating the I-SceI enzyme in cell cultures enriched at different cell cycle stages. By comparing the percentage of cells in a given cell cycle stage at the time of I-SceI activation against the HR outcome, we observed a strong positive correlation between the percentage of cells in S phase and the percentage of measured GFP^+^ HR events. As noted above, these experiments do not distinguish between S phase restriction of I-SceI-mediated breakage and S phase restriction of the HR pathway. However, given the known properties of I-SceI and precedent in the literature [11], [13], it is perhaps more likely that it is the engagement of HR itself that exhibits the observed cell cycle restriction. It will be valuable to re-examine this question in other cell types that can be synchronized in other ways. Indeed, the availability of this tightly regulated form of I-SceI will provide new opportunities to study interactions between the cell cycle, cell differentiation and mammalian DSB repair.

In view of the strong S phase linkage of I-SceI-induced HR in this system, treatments that strongly perturb HR without significantly affecting cell cycle distribution of the culture will likely affect HR during S phase. One such treatment, acute siRNA-mediated depletion of BRCA1, produced exactly these results, supporting the long-standing hypothesis that BRCA1 executes a major HR function during S phase [38].

## Supporting Information

Table S1 **Cell Cycle data and I-SceI-induced HR values for data shown in Figure 4B.**
(TIF)
