# Supplementary figures and images for: Cell Cycle-Dependent Induction of Homologous Recombination by a Tightly Regulated I-SceI Fusion Protein
Source: PLoS One. 2011 Mar 9;6(3):e16501. doi: 10.1371/journal.pone.0016501 (PMC3052302; doi:10.1371/journal.pone.0016501)

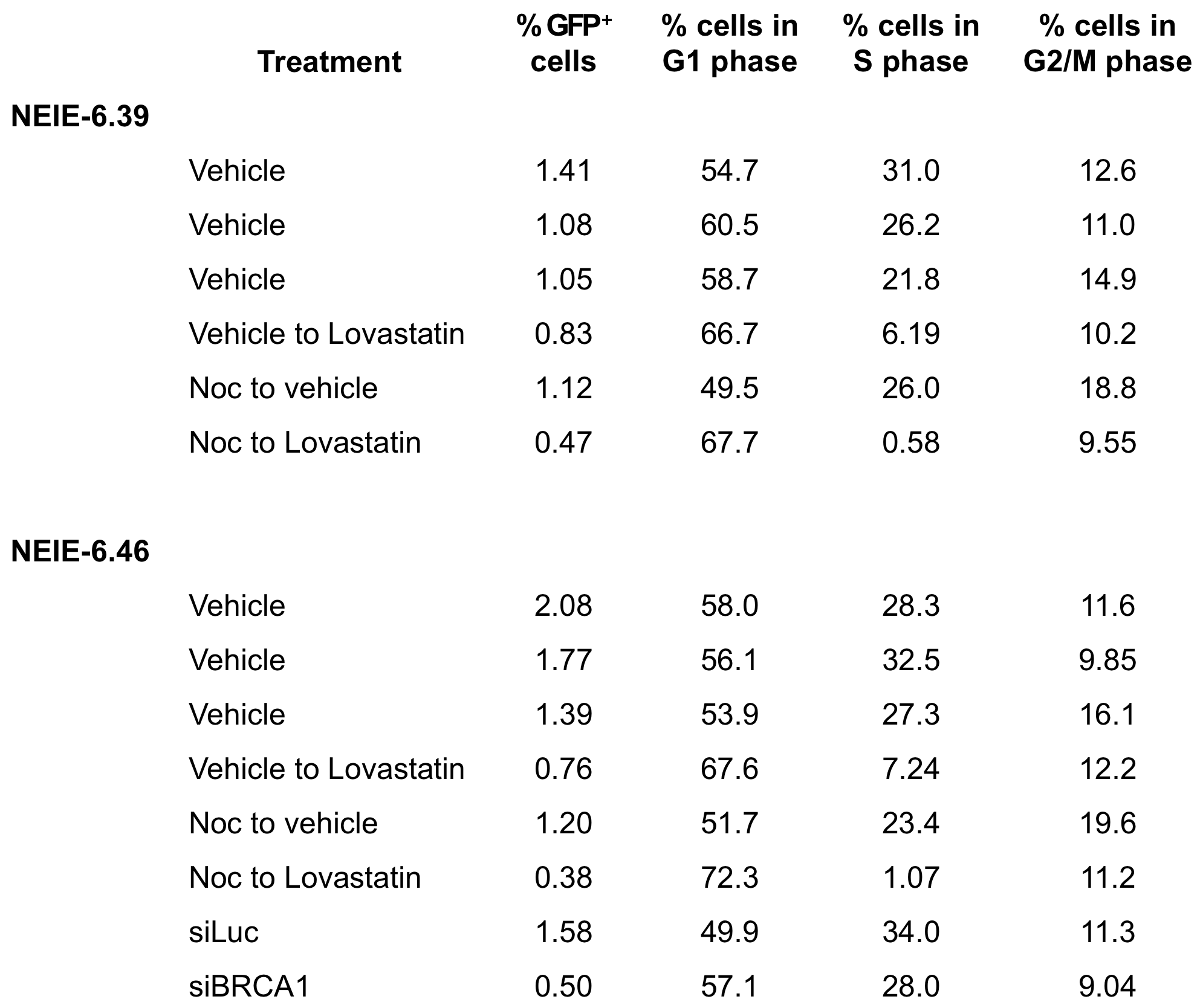

Supplement: Table S1 — Cell Cycle data and I-SceI-induced HR values for data shown in Figure 4B. (TIF) [file pone.0016501.s001.tif]
